# Red stripe on left forearm after stung by fish gill: A case report

**DOI:** 10.1097/MD.0000000000039849

**Published:** 2024-09-20

**Authors:** Ching-Hsiang Yu, Yu-Jang Su

**Affiliations:** a Department of Emergency Medicine, MacKay Memorial Hospital, Taipei, Taiwan; b Toxicology Division, Emergency Department, MacKay Memorial Hospital, Taipei, Taiwan; c Department of Nursing, Yuanpei University of Medical Technology, Hsinchu, Taiwan; d Department of Medicine, MacKay Medical College, New Taipei City, Taiwan.

**Keywords:** cellulitis, forearm, lymphangitis

## Abstract

**Rationale:**

Acute infectious lymphangitis represents a common complication of cellulitis, typically attributed to streptococcal infections after damaged skin integrity.

**Patient concern:**

This is a 51-year-old woman with a medical history of relapsing polychondritis, managed with steroid and methotrexate therapy in the outpatient department. She presented with a progressive redness and swelling of the left hand, accompanied by purulent discharge, persisting for 5 days. The patient had sustained a small cutting wound from the gill of a narrow-barred Spanish mackerel (Scomberomorus commerson) while cooking previously

**Diagnosis:**

Lymphangitis and cellulitis of the left forearm were diagnosed. A distinctive red streak was identified on the skin covering the palmaris longus muscle, consistent with the anatomical course of the median forearm lymphatic channel.

**Interventions:**

The patient received empirical intravenous ciprofloxacin (400 mg every 12 hours) and was subsequently admitted to the infectious disease ward. During hospitalization, the antibiotic regimen was adjusted to ceftazidime on the fourth day (2 g every 8 hours). The redness and swelling in the hand and arm gradually improved, and her blood culture showed no bacteria growth

**Outcomes:**

She was recovered and discharged on the seventh day with a prescription for oral clindamycin (150 mg every 6 hours).

**Lessons:**

A red stripe along the lymphatic route indicates acute lymphangitis and requires hospitalization for parenteral antibiotics.

## 1. Introduction

Skin, soft tissue infection is a commonly seen clinical problem in the emergency department. For the erythematous and swelling lesions, cellulitis, erysipelas, lymphedema, deep venous thrombosis and lymphoma were taken into the diagnostic lists.^[1]^ Sometimes the bacteria lead to gangrenous complications after coagulase-negative Staphylococcus infection in an skin, soft tissue infection.^[2]^

Acute lymphangitis is a not uncommonly seen inflammatory process involving the lymphatic system originating from a wound of a limb or body. It can be caused by bacterial infection, followed by parasitic infection (filariasis), mycobacterial infection, and malignancy (neoplastic lymphangitis).^[3]^

The tramlines sometimes spread with remarkable speed: within a few hours concomitant with symptoms including fever, chills, and malaise looking like bacteremia and sepsis. The diagnosis is distinct due to the characteristic linear erythematous streaks. The offended part most commonly involved was the upper extremity in 72% of patients, followed by the trunk in 18%, the lower extremity in 9%, and 1% over the abdomen in an India cases series report in 2023.^[4,5]^ The organisms that most commonly cause lymphangitis in individuals are gram-positive bacilli such as group A streptococci. In some animals, the bitten injury should be considered *Pasteurella multocida* or *Spirillum minus. Erysipelothrix, Mycobacterium marinum* in cases with fish or aquarium exposure.^[3,6–9]^ We reported a 51-year-old woman had red stripes on her left forearm after being stung by fish gills while cooking and also discussed some lymphangitis literature related to aquariums and fish.

## 2. Case Presentation

This is a 51-year-old woman with a medical history of relapsing polychondritis, managed with steroid and methotrexate therapy in the outpatient department. She presented with a progressive redness and swelling of the left hand, accompanied by purulent discharge, persisting for 5 days. The patient had sustained a small cutting wound from the gill of a narrow-barred Spanish mackerel (*Scomberomorus commerson*) while cooking previously. Despite initial wound treatment at a clinic, the wound failed to heal, and the redness and swelling extended from the middle finger to adjacent digits and further to the forearm. Although the patient denied fever, she reported experiencing chilliness, then she sought care at our emergency department.

**Figure 1.**
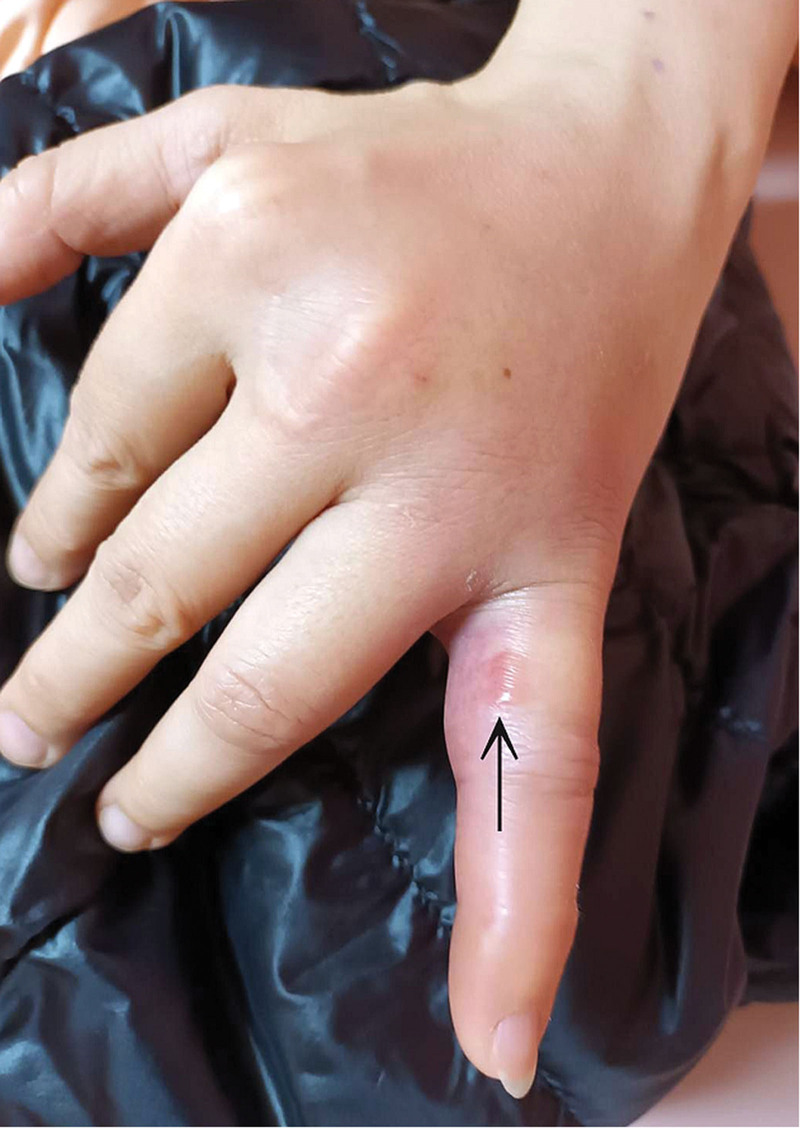
A small blister on the proximal phalanx of the left little finger (black arrow).

**Figure 2.**
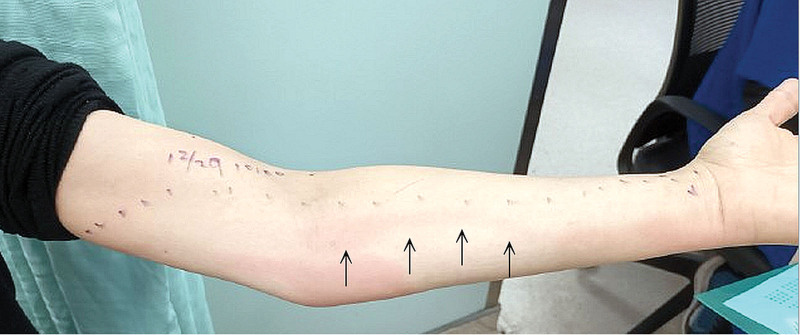
A linear red streak characterized by tenderness and swelling extending from the ulnar side of the wrist to the anterior side of the elbow (black arrow).

Upon arrival, her body temperature measured 37.9°C, with a heart rate of 88 beats per minute and a blood pressure reading of 128/70 mm Hg. The physical examination revealed a small blister on the proximal phalanx of the left little finger (Fig. 1, black arrow), along with a linear red streak characterized by tenderness and swelling extending from the ulnar side of the wrist to the anterior side of the elbow (Fig. 2, black arrow). Enlarged lymph nodes in the left axilla were also noted. Laboratory findings indicated an elevated white blood cell count (13,500/μL) and C-reactive protein level (11.0 mg/dL). The patient received empirical intravenous ciprofloxacin (400 mg every 12 hours) and was subsequently admitted to the infectious disease ward. During hospitalization, the antibiotic regimen was adjusted to ceftazidime on the fourth day (2 g every 8 hours). The redness and swelling in the hand and arm gradually improved, and her blood culture showed no bacteria growth, then the patient was discharged on the seventh day with a prescription for oral clindamycin (150 mg every 6 hours).

## 3. Discussion

Lymphangitis and cellulitis of the left forearm were diagnosed. A distinctive red streak was identified on the skin covering the palmaris longus muscle, consistent with the anatomical course of the median forearm lymphatic channel.^[10]^ It precisely differs from lymphedema because the circumference of limbs is not increased. Lymphedema can be either primary or secondary and secondary lymphedema is more common than primary lymphedema. The etiologies of secondary lymphedema are commonly seen as cancer, surgery, trauma, or infection.^[1]^

Acute infectious lymphangitis represents a common complication of cellulitis, typically attributed to streptococcal infections after damaged skin integrity. Given its lymphatic involvement, this inflammatory condition manifests as rapidly spreading erythematous streaks, with the potential to escalate into bacteremia or sepsis if timely intervention is not initiated. Lymphangitic streaking is distinguished by linear erythema aligning with involved lymphatic vessels and extending proximally toward regional lymph nodes.^[4]^ This distinctive cutaneous pattern serves as a valuable clinical indicator, aiding physicians in distinguishing lymphangitis from other soft tissue infections and facilitating the prompt administration of appropriate antibiotics or, if necessary, incision and drainage procedures.^[11]^ There are several reports about Mycobacterium spp. related lymphangitis listed in Table 1 and most of them are associated with contacting the aquarium.^[6–9]^

**Table 1:** Reported cases associated with fish and lymphangitis.

Year	Country	Age	Gender	Contact	Pathogen	References
2019	United States	69	Male	Admitted to cleaning an aquarium at home	Mycobacterium Marinum	[6]
2014	Spain	49	Male	Owned a fish aquarium	Mycobacterium marinum	[7]
2009	United States	61	Female	Cleaning her aquarium at home	Mycobacterium marinum	[8]
2007	Spain	37	Male	The bite of an aquarium fish	Mycobacterium haemophilum	[9]

In some severe lymphangitis cases may require topical or systemic steroids or even antibiotics when secondarily infected. Infective (bacterial, viral, fungal, or parasitic) lymphangitis is treated according to the type of infectious pathogens involved.^[4]^

## Author contributions

**Conceptualization:** Yu-Jang Su.

**Data curation:** Ching-Hsiang Yu, Yu-Jang Su.

**Formal analysis:** Ching-Hsiang Yu, Yu-Jang Su.

**Investigation:** Ching-Hsiang Yu, Yu-Jang Su.

**Methodology:** Yu-Jang Su.

**Project administration:** Yu-Jang Su.

**Resources:** Ching-Hsiang Yu, Yu-Jang Su.

**Software:** Yu-Jang Su.

**Supervision:** Yu-Jang Su.

**Validation:** Yu-Jang Su.

**Writing – original draft:** Ching-Hsiang Yu, Yu-Jang Su.

**Writing – review & editing:** Yu-Jang Su.
